# Evaluation of the Hybrid Tracer Indocyanine Green–^99m^Tc-Nanocolloid for Sentinel Node Biopsy in Bladder Cancer—A Prospective Pilot Study

**DOI:** 10.1097/RLU.0000000000004301

**Published:** 2022-06-18

**Authors:** Daphne D.D. Rietbergen, Erik J. van Gennep, Gijs H. KleinJan, Maarten Donswijk, Renato A. Valdés Olmos, Bas W. van Rhijn, Henk G. van der Poel, Fijs W.B. van Leeuwen

**Affiliations:** From the ∗ Interventional Molecular Imaging Laboratory, Department of Radiology; † Nuclear Medicine Section, Department of Radiology; ‡ Department of Urology, Leiden University Medical Center, Leiden; Departments of § Nuclear Medicine; ∥ Surgical Oncology, Netherlands Cancer Institute, Amsterdam, the Netherlands.

**Keywords:** bimodal tracer, bladder cancer, fluorescence-guided surgery, ICG-^99m^Tc-nanocolloid, interventional nuclear medicine

## Abstract

**Rationale:**

In muscle-invasive bladder cancer (MIBC), lymph node invasion has proven to be an independent predictor of disease recurrence and cancer-specific survival. We evaluated the feasibility of targeting the sentinel node (SN) for biopsy in MIBC patients using the hybrid tracer indocyanine green (ICG)–^99m^Tc-nanocolloid for simultaneous radioguidance and fluorescence guidance.

**Methods:**

Twenty histologically confirmed cN0M0 MIBC patients (mean age, 63.3 years; range, 30–82 years), scheduled for radical cystectomy with SN biopsy and extended pelvic lymph node dissection (ePLND), were prospectively included. Twelve patients were operated on following neoadjuvant chemotherapy. The patients received lymphoscintigraphy as well as SPECT/CT after 4 transurethral injections of ICG-^99m^Tc-nanocolloid (mean, 208 MBq; range, 172–229 MBq) around the tumor/scar in the detrusor muscle of the bladder on the day before radical cystectomy. Sentinel node resection was performed under radioguidance and fluorescence guidance.

**Results:**

Nineteen patients could be analyzed. On preoperative imaging, SNs could be identified in 10 patients (53%; mean, 1.6 SN/patient), which revealed drainage pathways outside the ePLND in 20% of the patients. Interesting to note is that 2 patients (10%) with preoperative nonvisualization displayed fluorescent and radioactive SNs during surgery. Location of the primary tumor near the left lateral side of the bladder seemed to be a factor for nonvisualization. Nodal harvesting with ePLND varied among patients (mean, 23.3). Histopathology confirmed tumor-positive nodes in 4 (21%) of all patients. In the 2 patients where an SN could be identified, the ePLND specimens were tumor-negative. All patients with tumor-positive nodes had advanced disease (stage III).

**Conclusion:**

Sentinel node biopsy in bladder cancer using the hybrid tracer ICG-^99m^Tc-nanocolloid is feasible, and preoperative imaging is predictive for the ability to perform SN biopsy in 83% of the patients who displayed an SN. In patients with a successful preoperative SN mapping using lymphoscintigraphy and SPECT/CT, the intraoperative SN guidance and detection were effective, even outside the ePLND area. As such, this study underscores the critical role that preoperative imaging plays in challenging image-guided surgery applications.

Bladder cancer is the seventh most diagnosed cancer in the male population worldwide and the 11th considering both sexes.^1^ In the Netherlands, almost 5000 patients are diagnosed with bladder cancer each year, which leads to more than 1200 deaths per year.^2^ Because of risk factors (eg, smoking, exposure to toxins, chronic irritation of the bladder), diagnostic strategies, and available treatment options, the incidence and treatment outcomes vary worldwide.^3^ For bladder cancer, lymph node invasion is an independent predictor and prognostic factor for disease recurrence and cancer-specific survival.^4^

Radical cystectomy (RC) is the treatment of choice for many patients with muscle-invasive bladder cancer (MIBC). This includes, according to the guidelines, a bilateral extended pelvic lymph node dissection (ePLND).^5–7^ Surgical planning for MIBC is mostly based on contrast-enhanced CT (CeCT) images of the abdominal and pelvic area. Unfortunately, this radiologic form of diagnostic imaging is limited in its capability to identify microscopic lymphatic spread. Because of the surrogate rule of the short axis on CeCT, CeCT can result in very low sensitivities (5%–54%) for the detection of nodal metastases.^8,9^ Approximately 25% of the cN0M0 MIBC patients who underwent an RC combined with ePLND have histopathologically proven metastatic lymph nodes,^10^ whereas preoperative CeCT fails to detect these nodal metastases in one-third of the patients. This suggests there is a discrepancy between the nodal status at presurgical staging and the metastatic lymph node burden seen in surgical samples. The molecular imaging modality ^18^F-FDG PET is becoming of more incremental value relevant in staging and recurrence disease because of the higher sensitivity compared with CeCT.^11^

An ePLND can increase the regional disease control but comes with increased comorbidities (eg, vascular, ureteral and nerve injury, lymphocele, thromboembolic events) and will miss nodal metastases outside the resection template. Sentinel node (SN) biopsy can provide a less invasive and more personalized lymph node (LN) dissection. The SNs that usually are the first site of tumor seeding, preceding systemic spreading,^12–17^ can be preoperatively identified by mapping—via lymphoscintigraphy and SPECT/CT—the drainage profile of a radiocolloid. This procedure was initially described for bladder cancer by Sherif et al.^12^ Intraoperative SN identification was realized by complementing the preoperative imaging roadmap with the use of gamma probe to identify the radiocolloids. Alternative means for intraoperative SN identification have been pursued using blue dye and/or the fluorescence dye indocyanine green (ICG).^12–24^

In the present study, we have used the hybrid tracer ICG-^99m^Tc-nanocolloid in patients with MIBC who underwent RC with or without neoadjuvant chemotherapy (NAC). This hybrid tracer has successfully been used for SN biopsy in various malignancies^25^ but, so far, remained undocumented in bladder cancer. To establish its feasibility, we compared the results of the preoperative SPECT/CT-based SN mapping with the intraoperative SN identification using complementary radioguidance and fluorescence guidance.

## PATIENTS AND METHODS

### Patient Demographics

This prospective trial was registered at the Netherlands Trial Register as NL48901.031.14 and was executed at the Netherlands Cancer Institute–Antoni van Leeuwenhoek Hospital after approval of the Medical Ethical Review Board of the institution (M14HSN). Patients with histologically confirmed MIBC or high-risk patients (carcinoma in situ) were scheduled for SN biopsy before RC combined with ePLND. Patients were preoperatively staged using CT–intravenous urogram and ^18^F-FDG PET for imaging and underwent transurethral tumor resection. Half of these patients received NAC. All patients were prospectively included after informed consent. The main exclusion criteria were suspected nodal disease on imaging and prior pelvic radiation and/or surgery.

### SN Imaging Procedure

**FIGURE 1:**
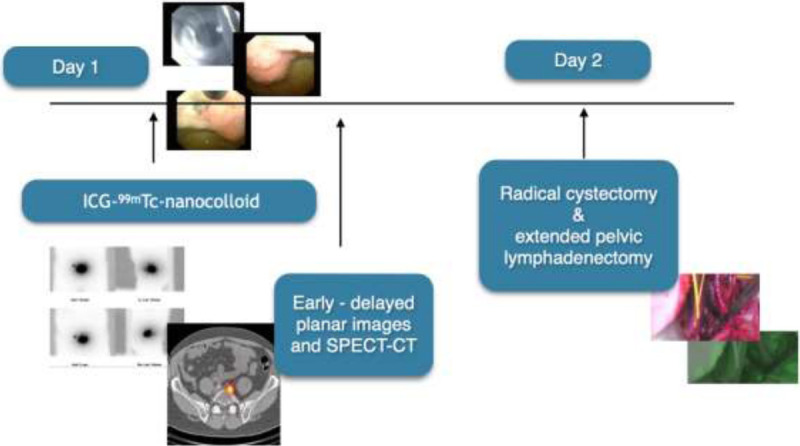
Flowchart of the study design. On the first day, 208 MBq (SD, 15.8; 5.6 mCi) ICG-^99m^Tc-nanocolloid was injected in 4 to 6 transurethral injections into the detrusor muscle of the bladder; around the tumor, divided over the bladder. The procedure took place under cystoscopic guidance using an endoscopic needle. Lymphatic drainage was mapped using early and delayed static lymphoscintigraphy (15 minutes and 2 hours postinjection) and SPECT/CT (2 hours postinjection). On the second day, the patient was operated during open or robotic (DaVinci) procedure. The SNs were intraoperatively identified under combined radioguidance and fluorescence guidance using a (laparoscopic) gamma probe and fluorescence camera (Firefly or Hamamatsu for robotic and open surgery, respectively).

**FIGURE 2:**
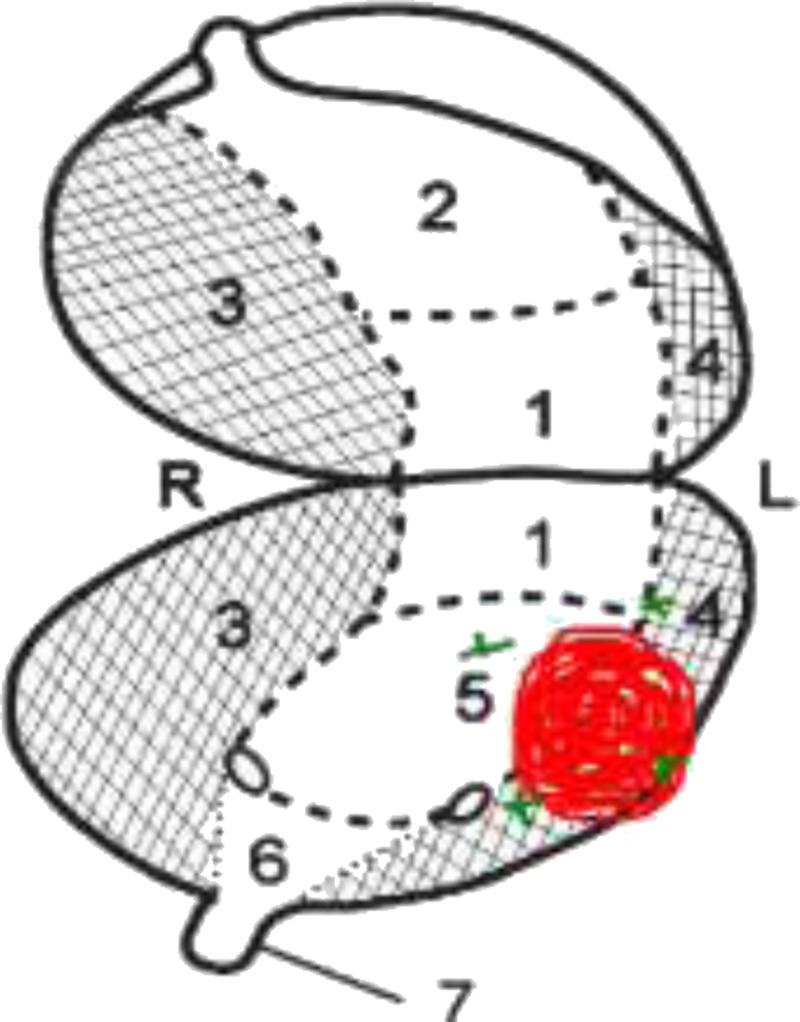
Graphic image of the bladder with its corresponding zones (1. dorsal part, 2. apex vesicae, 3. base, 4. left lateral border, 5. right lateral border, 6. trigonum vesicae). Red dot corresponds with the location of the tumor in this particular case; the surrounding green crosses correspond with the tracer injection site.

All patients followed a 2-day SN procedure. On the day before surgery, a volume of 2 mL containing 208 MBq (SD, 15.8; 5.6 mCi) ICG-^99m^Tc-nanocolloid (GE Healthcare, Leiderdorp, the Netherlands) was injected in 4 transurethral injections into the detrusor muscle of the bladder, around the tumor or transurethral resection scar, under cystoscopic guidance using an endoscopic needle (Injetak; Laborie) with a 5-mm tip length. Lymphatic drainage was mapped based on early and delayed static lymphoscintigraphy (15 minutes and 2 hours postinjection) as well as SPECT/CT (2 hours postinjection) using a dual-head SPECT/CT gamma camera (Symbia T6; Siemens, Erlangen, Germany) equipped with a 6-row CT scanner. SPECT parameters were as follows: 128 × 128 matrix, zoom of 1.0, and 180-degree rotation with 20 views per head (30 seconds per view). CT (130 keV, 40 mAs, B30s kernel, 2-mm axial reconstruction) was used for attenuation correction and anatomical localization. Multiplanar reconstruction, image fusion, and 3D image reconstruction of the SPECT/CT images were done using an Osirix software program (Pixmeo SARL, Bernex, Switzerland). Planar and SPECT/CT images were evaluated with respect to drainage to lymph node basins, SN visualization, and non-SN uptake and their corresponding anatomical localizations. Nodes were considered as SN when visualized as intense focal uptake on the early, delayed images and/or SPECT, with increase in uptake in time. When SNs were visualized, SPECT/CT images were used to indicate their anatomical location in relation to muscles and vessels in the draining lymph node basins. A flowchart of the study setup and a graphic image of the bladder with its corresponding anatomical zones are presented in Figure 1 and Figure 2, respectively.

### Surgical Guidance

The day following tracer administration and imaging, the patient was operated on using DaVinci Surgical Robotic System (Intuitive Surgical, Sunnyvale, Calif) or underwent an open RC. The SNs were intraoperatively identified under combined radioguidance and fluorescence guidance using a laparoscopic gamma probe (Neoprobe; Johnson & Johnson Medical, Hamburg, Germany) and an integrated fluorescence camera (Firefly) in robotic surgery; a traditional gamma probe (Europrobe; Eurorad, Strasbourg, France) and FIS-00 Hamamatsu camera (Hamamatsu Photonics K.K., Hamamatsu, Japan) were used in open setting. Excised samples were also examined ex vivo by using the FIS-00 Hamamatsu camera.

### Pathology

All surgically removed nodes (SNs and non-SNs in separate packages) were histopathologically examined for tumor cells by the pathologist. Micrometastases were defined as lesions smaller than 2 mm and macrometastases as lesions larger than 2 mm, whereas conglomerates of isolated tumor cells smaller than 0.2 mm were denoted as submicrometastases.

## RESULTS

Twenty patients (mean age, 63.3 years; range, 30–82 years) were included for the preoperative SN procedure. One patient was operated on in another hospital and was excluded from analysis. In total, 19 patients were evaluated. Patient characteristics are displayed in Table 1. Twelve patients (63%) were pretreated with NAC, and 7 (37%) patients did not receive treatment before surgery. Seven patients underwent an open RC, and 12 patients were operated on using DaVinci Surgical robot.

**TABLE 1:** Schematic Overview of the Results

Patient (Sex, Age)	Primary Lesion Site and Region	Surgery	NAC/NAC-Naive	SLN SPECT/CT	Side	Non-SN	Basin	Lymph Node OR	ePLND No. Nodes	T at Pathology	Nodes at Pathology
1 (F, 82 y)	Rt, 3	O	No NAC	1	Rt	0	Obt	1	39	3b	Positive
2 (M, 55 y)	Lt, 5	R	NAC	1 1	Rt Lt	0	Obt ext	3	13	3a	Positive
3 (M, 77 y)	Lt, 4	R	No NAC	0	—	0	—	0	16	2	Negative
4 (M, 47 y)	Rt, 3	R	NAC	1	Rt	0	Obt	8	40	0	Negative
5 (M, 56 y)	Lt, 5	O	NAC	0	—	0	—	6	15	3b	Positive
6 (M, 74 y)	Lt, 4	O	NAC	1	Lt	0	Obt	0	10	CIS	Negative
7 (F, 72 y)	Lt, 2–4	R	No NAC	0	—	0	—	0	9	CIS	Negative
8 M, 42 y)	Lt, 4	R	NAC	0	—	0	—	0	19	0	Negative
9 (M, 64 y)	Rt, 1–3	R	NAC	1 1	Rt Lt	1	Obt ext	7	17	2b	Negative
10 (M, 73 y)	Lt, 1–4	O	NAC	0	—	0	—	0	41	3a	Negative
11 (M, 70 y)	Lt, 4	R	NAC	0	—	0	—	0	29	CIS	Negative
12 (M, 79 y)	Lt, 4	R	No NAC	0	—	0	—	1	22	2b	Negative
13 (F, 30 y)	Rt, 2	O	NAC	3 1	Rt Lt	3	Ext (2×), obt obt	2	31	0	Negative
14 (M, 58 y)	Lt, 4–5	R	NAC	1	Lt	0	Com	1	32	CIS	Negative
15 (M, 62 y)	Lt, 3–5	O	No NAC	0	—	0	—	1	29	1	Negative
16 (M, 82 y)	Rt, 2–3	O	No NAC	1	Rt	0	Com/aor	2	21	1	Negative
18 (F, 49 y)	Rt, 3	R	No NAC	1	Rt	0	Obt	1	11	CIS	Negative
19 (M, 60 y)	Lt, 4	R	NAC	0	—	0	—	0	33	3b	Positive
20 (M, 71 y)	Lt, 4	R	NAC	1 1	Rt Lt	0	Com com	3	15	3a	Negative
n = 19 Mean age, 63 y Range, 30–82 y				n = 16 SN		n = 4 non- SN	n = 15 basins				SN Positive = 4/19 patients. (21%)

Of the 20 patients who were included, 19 patients could be evaluated. In 52.6% (n = 10 patients), lymphoscintigraphy (SPECT/CT) reveals at least 1 SLN. Of those patients (visualized SLN), 6 patients revealed 1 SLN (60%); in 3 patients, 2 SLNs were seen (30%), and in 1 patient, 4 SLNs were seen. During surgery, all except 1 marked SLNs were seen. Histopathology-positive nodes containing metastases were seen in 4 patients (21%), and in 8 patients, these nodes were without metastases. In 9 patients (47.4%), a nonvisualization was seen on preoperative imaging; in some cases (3/9), the surgeon could find a lymph node during surgery, and in 6 of 9 patients, the surgeon did not find a lymph node either. Pathology of the lymph nodes did not reveal any metastasized disease.

1, Dorsal; 2, apex vesicae; 3, base; 4, left lateral border; 5, right lateral border; aor, aortic; CIS, carcinoma in situ; com, common iliac artery; ext, extern iliac artery; F, female; Lt, left; M, male; noNAC, NAC-naive patients; O, open; obt, obturator; R, robotic; Rt, right.

### Sentinel Nodes

**FIGURE 3:**
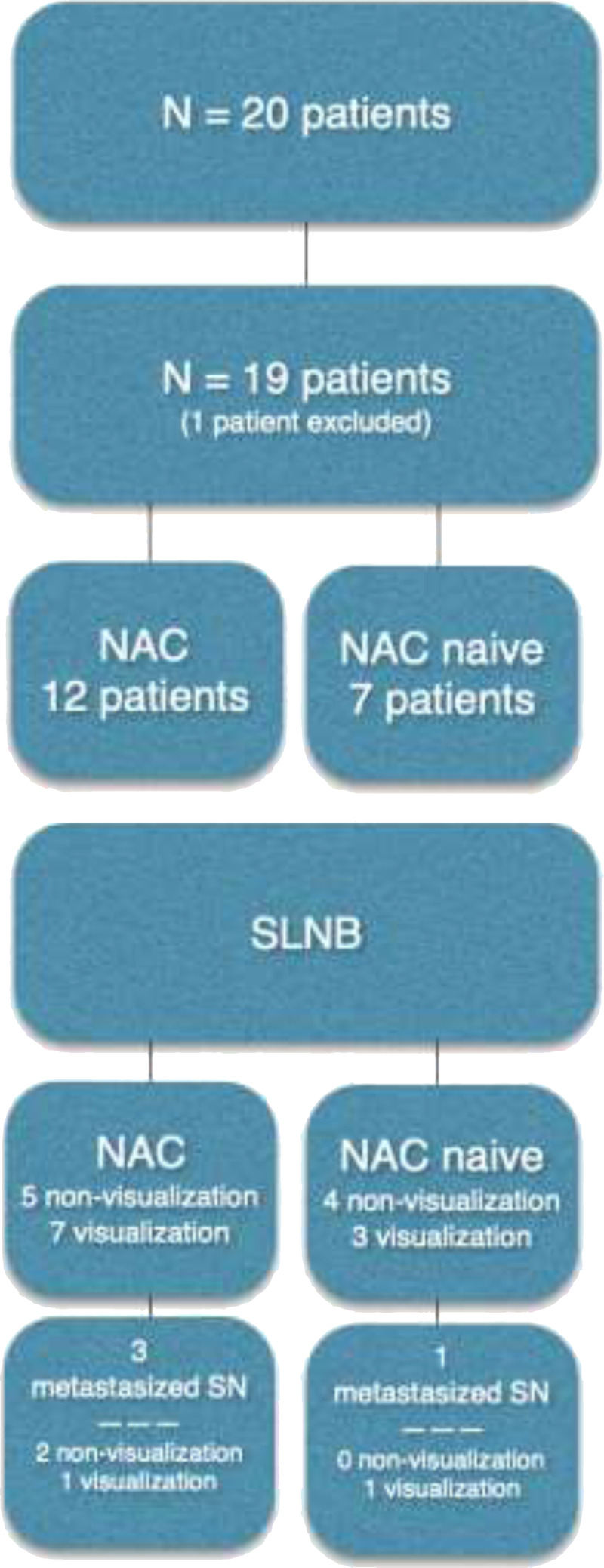
Flowchart of the study, which included 20 patients. One patient was operated on in another hospital and had to be excluded from analysis, resulting in a group of 19 patients analyzed. Twelve patients in the NAC group and 7 patients in the NAC-naive group. All patients underwent an SN procedure.

In 53% (n = 10 patients; 7 in the NAC group and 3 in the NAC-naive group), lymphoscintigraphy and SPECT/CT revealed SN(s). A single SN was found in 6 patients (60%), whereas 2 SNs could be identified in 3 patients (30%), and in 1 patient, 4 SNs were observed (10%) in 1 patient. In the remaining 47% of the cases (n = 9 patients; 5 patients in the NAC group and 4 patients in the NAC-naive group, respectively), no SNs could be visualized before surgery. A flowchart of the study setup is presented in Figure 3.

In 9 of the 10 patients with SNs at preoperative imaging, in total 16 SNs could be resected by complementing the preoperative imaging roadmap with intraoperative radioguidance and fluorescence guidance. In 1 robot-operated patient with early-stage tumor of the left lateral border of the bladder, the SN could not be identified during surgery, and ePLND also did not reveal any metastatic disease. The number of additional nodes that were resected during ePLND varied among patients (mean, 23.3; range, 9–41). In the 9 patients without SN visualization on preoperative imaging, 2 radioactive or fluorescent nodes were detected during surgery. This means that overall intraoperative SN guidance was successful in 63% of the patients.

### Relation Between Primary Tumor Location and Preoperative SN Visualization

**FIGURE 4:**
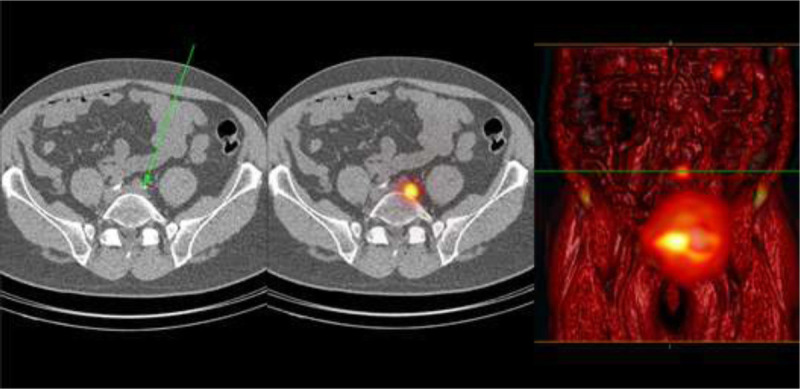
A blurry image is seen without an obvious focus or enlarged lymph node on SPECT/CT images, shine-through phenomena with nonvisualization of the SN, where the injection site shines through and could outshine possible SN nearby. This could lead to false-negative findings.

For tumors located in the base or apex of the bladder, 100% SN visualization was obtained (Fig. 4). Of the patients displaying nonvisualization on preoperative lymphoscintigraphy, in 56% the primary tumor was located at the left lateral side (n = 5).

The tumors of the 2 patients with preoperative nonvisualization and a positive SN detection during surgery were located at the right side and base/right lateral border, respectively. Other sides with nonvisualization as a result corresponded with apex/left lateral (n = 1), dorsal/left lateral (n = 1), base/right lateral (n = 1), and right lateral border (n = 1).

### Location of SNs in Relation to ePLND Template

**FIGURE 5:**
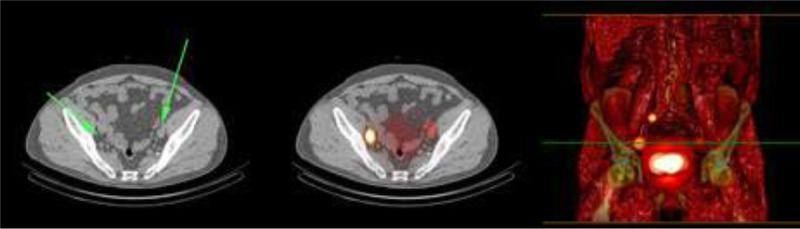
On the left, CT image of the same area revealed no pathologic enlarged lymph node at the right side (arrow right). At the left side, an enlarged lymph node is seen (arrow left). In the middle, transaxial fused SPECT/CT image of lymphatic drainage with SN seen along the iliac artery at both sides. On the right, volume-rendered fusion image of both scintigraphy SPECT and CT is seen with focal uptake in the SN along the right iliac artery. At the left side, a contralateral SLN is seen (crossover phenomenon). At the right side, a higher echelon node is seen along the common iliac artery.

**FIGURE 6:**
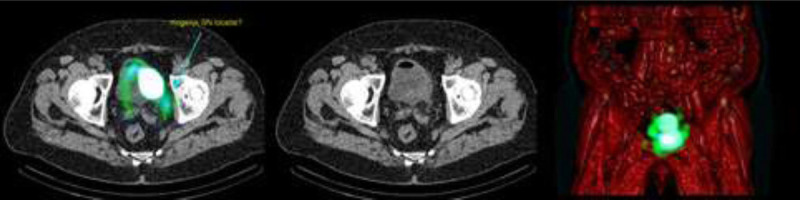
Upper row: Fused SPECT/CT, CT, and volume-rendered fusion image SPECT. Fused images show a focal radioactive signal in the right obturator foramen, marked as the SN. The green arrow on CT shows the corresponding nonenlarged lymph node. Lower row: left: Intraoperative view with white light, right: view of the fluorescent camera. White arrow shows focal ICG uptake and the corresponding lymph node, the SN.

**FIGURE 7:**
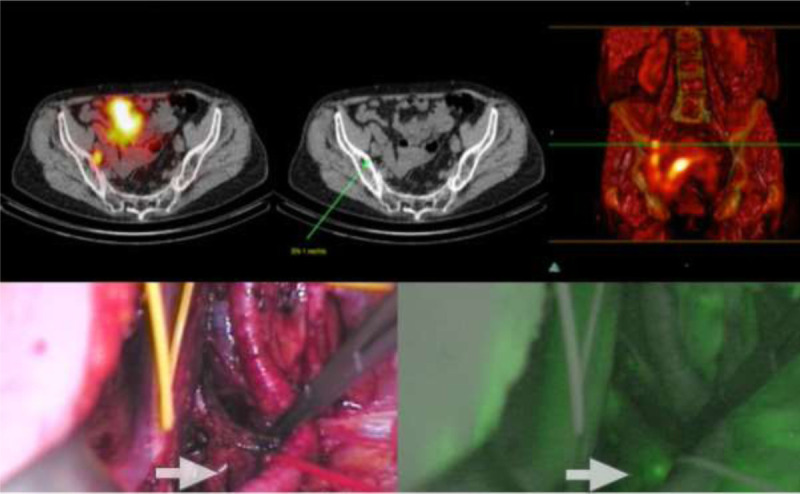
In the middle, transaxial fused SPECT/CT image of lymphatic drainage with SN seen above the normal ePLND area, in the aortic region. On the left, CT image of the same areas revealed a nonpathologic enlarged lymph node (arrow). On the right, volume-rendered fusion image of both scintigraphy SPECT and CT.

Of the visualized SNs, 80% were found within the ePLND template, 53% in the obturator fossa and 27% at the external iliac artery (Figs. 5-6). In 3 patients (20%), SNs were found outside the ePLND template and 1 aberrant SN localization above the ureteroiliac vessels crossing and the others along the common iliac artery or aorta (Fig. 7). Bilateral drainage was seen in 40% of the patients with preoperative imaging. In the remaining 60% of the patients, including the only midline tumor, unilateral draining was observed.

### Histopathology

In total, 4 of the 19 patients' (21%) histopathology showed lymph node metastases: 2 in the visualization and 2 in the nonvisualization group, respectively. All tumor-positive nodes, either seen in SN or ePLND material, concerned advanced disease (Fig. 3 and Table 1). In all cases with SN visualization on preoperative imaging, the ePLND specimens were tumor-negative, where the SN was the only localization that contained metastatic disease. No additional (micro)metastatic disease was seen in patients with <pT3 tumor(s). In the nonvisualization group, 2 patients (NAC group) had a tumor-positive node in their ePLND specimens, both concerning locally advanced pT3–4 disease.

### Neoadjuvant Chemotherapy

Patients pretreated with NAC showed less nonvisualization on preoperative scintigraphy. On the other hand, those patients were more involved in metastasized nodal disease: 3 patients versus 1 patient in the NAC-naive group.

## DISCUSSION

In this pilot study, we demonstrated the feasibility of the hybrid tracer ICG-^99m^Tc-nanocolloid for SN biopsy in clinical node-negative (cN0) MIBC patients scheduled for RC with ePLND. In 53% of the patients, the preoperative SN procedure was successful. Despite having a preoperative nonvisualization, an additional 10% of the patients displayed an SN during surgery. This means that overall preoperative imaging roadmaps were predictive for the utility of image guidance in 63% of the patients who displayed an SN. The degree of lymphatic drainage of the bladder seemed to be influenced by the location of the primary tumor, and unpredictable drainage and drainage outside the ePLND template were seen in 20%.

Previous studies have explored the feasibility of SN mapping in bladder cancer patients using radioactive colloid, blue, and fluorescent dyes as separated modalities.^13–21^ Unfortunately, most studies are based on relatively small patient groups. The studies are also not mutually comparable to ours because of differences in the injection technique and type of tracer that was used. Besides that, there is also a difference in the implementation of preoperative imaging data and the type of intraoperative imaging modalities used. From a surgical perspective, optical SN identification is considered highly desirable. This can be realized using, for example, blue dye or “free” ICG. Relying on such optical agents only for guidance, however, means that the procedure cannot receive guidance via preoperative imaging roadmaps. Also, as a result of their molecular size, these lymphangiographic agents facilitate more rapid drainage in comparison to radiocolloids, but lack SN specificity.^26,27^ In line with this, studies relying solely on free ICG reported higher numbers of SN compared with the 1.6 SN per patient reported by us.^15^ This difference in flow rates has also an impact on bilateral drainage patterns. We saw a bilateral drainage in 40% of the patients with preoperative imaging, which is in line with the literature of radiocolloid-based SN localization.^14^ However, studies using only free ICG reported a much higher bilateral drainage pattern (90%). Arguing that in pursuit of precision surgery “less is more,” one can argue that use of lymphangiographic rather than SN-specific agents means the procedure drifts away from its original minimally invasive concept. Using hybrid tracers such as ICG-^99m^Tc-nanocolloid intraoperative optical imaging can be combined with SN specificity and preoperative imaging.^25,28–31^

In our study, the preoperative SN visualization rate on a per-patient basis was 53%, although lower than other SN applications, this value is in line with the 23% to 94% range described in the literature.^12–17^ As seen in the study by Liedberg et al,^18^ omitting the preoperative imaging, the percentage of SN identification dropped to 23%. We found that preoperative nonvisualization was highly related to the chance of intraoperative SN identification. Intraoperative detection rate of preoperatively defined SN using the gamma probe in our study was 90%, which was in line with the reported 81% to 100% in the literature.^13,14,16,18–21^ Using fluorescence imaging only, we could detect 58%, which was in the high end if compared with the detection percentages reported for use of ICG only (0%–90%).^22–24^

Histopathology-confirmed tumor-positive SNs were seen in 21% of the total population, which is in line with earlier findings in the literature, although Polom et al^14^ reported 34% nodal metastases in their resected SNs. In the study by Polom et al,^14^ 6.4% of the SNs were seen outside the PLND area. The radiotracer study by Liedberg et al^18^ found more than 40% outside the obturator foramen. In our study, unexpected localizations were found in 20% and concerned mostly patients from the NAC group (66%), independent of tumor stage, indicating that the neoadjuvant treatment could alter lymphatic drainage.

The current study is limited by the sample size, heterogeneity between patients, and lack of outcome data. Also, the SN nonvisualization rate of 47% reported by us and others represents a serious limitation concerning the added value of SN in MIBC. This suggests that there is room for improvement concerning the ability to induce lymphatic tracer drainage from primary bladder cancer. Hence, a more extensive research based on a more homogenous group of patients is needed to show whether SN biopsy adds clinical value by staging the pelvis in bladder cancer. Interestingly, correlating the location of the primary tumor and injection side and the ability of successful SN procedure revealed a higher nonvisualization in the tumors located at the left lateral border compared with other localizations. Future studies need to focus on methodological aspects related to bladder pressure,^14,22^ stimulation of lymphatic flow, and standardization of injection techniques^32^ to increase SN visualization rates.

## CONCLUSION

In cN0M0 MIBC patients, the SN procedure using ICG-^99m^Tc-nanocolloid seems feasible with SN visualization in 63% of the patients (53% preoperative and 58% intraoperative). In patients with a successful preoperative SN mapping using lymphoscintigraphy and SPECT/CT, the intraoperative SN guidance and detection were effective, even outside the ePLND area.
